# Isolation of Highly Suppressive CD25+FoxP3+ T Regulatory Cells from G-CSF-Mobilized Donors with Retention of Cytotoxic Anti-Viral CTLs: Application for Multi-Functional Immunotherapy Post Stem Cell Transplantation

**DOI:** 10.1371/journal.pone.0085911

**Published:** 2014-01-17

**Authors:** Edward R. Samuel, Lorea Beloki, Katy Newton, Stephen Mackinnon, Mark W. Lowdell

**Affiliations:** 1 Department of Haematology, University College London, Royal Free Campus, London, United Kingdom; 2 Oncohematology Research Group, Navarrabiomed-Miguel Servet Foundation, Pamplona, Spain; 3 Cell Medica Ltd, and University College London, London, United Kingdom; 4 Department of Haematology, University College London and The Royal Free London NHS Foundation Trust, London, United Kingdom; New York University, United States of America

## Abstract

Previous studies have demonstrated the effective control of cytomegalovirus (CMV) infections post haematopoietic stem cell transplant through the adoptive transfer of donor derived CMV-specific T cells (CMV-T). Strategies for manufacturing CMV immunotherapies has involved a second leukapheresis or blood draw from the donor, which in the unrelated donor setting is not always possible. We have investigated the feasibility of using an aliquot of the original G-CSF-mobilized graft as a starting material for manufacture of CMV-T and examined the activation marker CD25 as a targeted approach for identification and isolation following CMVpp65 peptide stimulation. CD25+ cells isolated from G-CSF-mobilized apheresis revealed a significant increase in the proportion of FoxP3 expression when compared with conventional non-mobilized CD25+ cells and showed a superior suppressive capacity in a T cell proliferation assay, demonstrating the emergence of a population of Tregs not present in non-mobilized apheresis collections. The expansion of CD25+ CMV-T in short-term culture resulted in a mixed population of CD4+ and CD8+ T cells with CMV-specificity that secreted cytotoxic effector molecules and lysed CMVpp65 peptide-loaded phytohaemagglutinin-stimulated blasts. Furthermore CD25 expanded cells retained their suppressive capacity but did not maintain FoxP3 expression or secrete IL-10. In summary our data indicates that CD25 enrichment post CMV stimulation in G-CSF-mobilized PBMCs results in the simultaneous generation of both a functional population of anti-viral T cells and Tregs thus illustrating a potential single therapeutic strategy for the treatment of both GvHD and CMV reactivation following allogeneic haematopoietic stem cell transplantation. The use of G-CSF-mobilized cells as a starting material for cell therapy manufacture represents a feasible approach to alleviating the many problems incurred with successive donations and procurement of cells from unrelated donors. This approach may therefore simplify the clinical application of adoptive immunotherapy and broaden the approach for manufacturing multi-functional T cells.

## Introduction

Cytomegalovirus (CMV) reactivation continues to be a significant cause of morbidity and mortality following allogeneic haematopoietic stem cell transplantation (aHSCT) [1], [2] with the incidence of CMV disease being reported to be as high as 70% [3]. Several strategies have been employed in the manufacture of donor-derived CMV-specific T cells (CMV-T) for adoptive transfer over the last two decades that have successfully demonstrated both safety and efficacy in restoring antiviral immunity [4]–[10]. More recently the direct selection of γ-secreting (IFN-γ) cells in response to CMVpp65 peptide stimulation [11], [12] has simplified generation of CMV-T, significantly reduced the manufacturing time and has also been successfully used to select T cells specific for adenovirus (AdV) and Epstein Barr virus (EBV) for clinical use [13], [14]. Isolation of antigen-specific T cells through the identification of activation markers that are up-regulated after T cell activation is also a promising alternative. T cell activation markers offer an increased sensitivity over approaches such as IFN-γ secretion as they are independent of cytokine secretion and therefore could allow the isolation of increased numbers of antigen-specific T cells. Several T cell activation markers have been identified including CD25, CD69, CD137 and CD154 [15]–[20] with differing temporal dynamics that allow for simultaneous detection and enrichment. The availability of good manufacturing practice (GMP) compliant CD25 antibodies for clinical use makes the selection of CMV-T through CD25 selection a feasible option. Indeed several groups have investigated CD25 in large scale clinical manufacture for potential use in adoptive transfer [21], [22] due to the commercial availability of CD25 reagents.

To date models for CMV-T manufacture have focussed primarily on using peripheral blood mononuclear cells (PBMCs) collected by leukapheresis from the original HSCT donor. The procurement of an additional apheresis for CMV-T manufacture is associated with some degree of difficulty especially in the unrelated donor setting where donor refusal, registry refusal and scheduling difficulties can prevent collection. The prospect of manufacturing antigen-specific T cells from an aliquot of the original HSCT obtained by leukapheresis after mobilization by recombinant human granulocyte-colony stimulating factor (G-CSF) as an alternative PBMCs source is attractive. Murine and human studies have suggested that G-CSF-mobilization inhibits type 1 cytokine production by T cells, through inhibition of secretion at a single cell level as well as reducing the fraction of cytokine-secreting cells in the periphery, arguing against the use of these cells for adoptive immunotherapy [23]–[27]. Furthermore extensive gene expression profiling in G-CSF-mobilized PBMCs has revealed the up-regulation of genes related to T helper cells type 2 (TH_2_) and Treg cells and down-regulation of genes associated with T helper cells type 1 (TH_1_), cytotoxicity, antigen presentation and GvHD [28]. However there has been little published data with regard to the effect of G-CSF on the anti-viral T cell response and the influence of G-CSF in this regard, beyond the time of apheresis. The clinical use of G-CSF-mobilized donor lymphocytes administered for therapy of relapse post aHSCT in acute myeloid leukaemia (AML) has demonstrated efficacy with a similar graft versus leukaemia (GvL) response when compared with conventional non-mobilized donor lymphocytes [29], [30]. These results alleviate some of the major concerns surrounding the feasibility of using G-CSF-mobilized lymphocytes as a starting material for the manufacture of anti-viral immunotherapies. We have published our previous findings that demonstrated the feasibility of employing the use of G-CSF-mobilized PBMCs as an effective strategy for manufacture of CMV-T with the retention of functional CMV-specific cytotoxicity comparable with non-mobilized PBMCs, using CD154 based selection [31]. But the translation of this particular approach is currently restricted by the non-availability of a GMP-compliant anti-CD154 antibody. More recently the generation and adoptive transfer of CMV-T cell lines expanded from G-CSF-mobilized PBMCs in short term culture has been demonstrated in a phase I/II clinical trial, where seven patients received CMV-T cell products [32]. However, the widespread application of short-term culture manufacturing processes can be complex to translate into cellular therapy laboratories, from a both a regulatory and GMP compliant perspective.

In this study we explore the feasibility of using CD25-based enrichment of CMV-T from G-CSF-mobilized PBMCs due to the relative simplicity and rapidity of current direct selection methods. Identification of antigen-specific T cells through CD25 expression can be confounded by the similar CD25 expression pattern exhibited by regulatory T cells (Tregs). Tregs are a subset of CD4+ T cells that are suppressive in nature and regulate responses towards tumour, foreign and allo-antigens that constitutively express CD25 [33], [34]. The transcription factor forkhead box P3 (FoxP3) has also been identified to play a role in the development and function of Tregs and is also used as a phenotypic marker of Tregs [35]. Ukena and colleagues [36] in their studies report the application of G-CSF resulted in a significant increase in Treg yield and that cells retain a cytokine profile and phenotype associated with Treg characteristics and remained highly suppressive. Previous reports in non-mobilized PBMCs have identified populations of both antigen-specific T cells and Tregs following CD25 enrichment and argue that the adoptive transfer of both these populations in a single immunotherapy product could prove beneficial to recipients of aHSCT who are at risk of both CMV reactivation and GvHD [21].

We have therefore investigated the phenotype and function of CMV-T isolated through CD25 expression from G-CSF-mobilized PBMCs with regard to FoxP3 expression and suppressive capacity and explored the impact of removal of CD4+ CD25+ Tregs from starting populations to augment selectivity of antigen-specific T cells for adoptive immunotherapy. CD25+ CMV-T were expanded in short term culture and subsequently analysed for both CMV-specificity through cytokine profiling and cytotoxicity together with suppressive function and FoxP3 expression to ascertain the retention of Tregs.

## Materials and Methods

### Blood Donors and Cell Preparation

Prior to sample collection ethical approval was given by the National Health Service (NHS) Health Research Authority (North West London Research Ethics Committee) and permission obtained from the institutional research and development office of Royal Free London Foundation Trust. After obtaining signed written informed consent, fresh blood samples were taken from both G-CSF-mobilized and non-mobilized CMV-seropositive healthy donors, after a 3–5 hour collection on the COBE Spectra apheresis system (Caridian BCT, Lakewood, CA). PBMCs were isolated from all donor samples after density gradient separation using Lymphoprep (Axis Shield Diagnostics, Dundee, UK) and subsequently cultured in RPMI 1640 Medium supplemented with 1% antibiotic (Both Life Technologies, Paisley, UK) and 10% heat inactivated human AB serum (Biosera, Ringmer, UK) at a concentration of 1×10^7^/ml. PBMCs were stimulated for up to 24 hours in flat bottom 6 well culture plates (NUNC, Roskilde, Denmark) with 1 µg/peptide/ml of CMVpp65 peptide pool spanning the entire CMVpp65 protein (CMVpp65 Peptivator. Miltenyi Biotec, Bergisch Gladbach, Germany) and incubated at 37°C/5% CO_2_. In some experiments freshly isolated PBMCs were cryopreserved at 1∶1 with human serum albumin (HSA) 4.5% (Bio Products Laboratory Ltd, Elstree, UK) containing 20% DMSO (WakChemie, Steinbach, Germany) according to standard protocols, for use in future experiments as well as a source of feeder cells.

### Flow Cytometric Analysis

Flow cytometry experiments consisted of four to six colour panels where a minimum of 50,000 CD3+ events were acquired after gating of viable lymphocytes using FSC and SSC signals on a FACScan flow cytometer (Cytek UK) and data analysed using FlowJo version 7.6 (TreeStar Inc. Ashland, OR). For control staining of cytokines and activation markers we used PE-conjugated mouse antibodies of matching isotype and supplier of the retrospective antibodies. Cells were stained for 15 minutes in the dark, washed in 2 ml of HBSS for 5 minutes and resuspended in 200 µl of FACS Flow (BD Bioscience, Franklin Lakes, NJ) before acquisition.

### Time Course Assay of Activation Marker Kinetics

PBMCs isolated from mobilized and non-mobilized donors were stimulated in 96 well plates at a concentration of 1×10^7^/ml for 24 hours with either CMVpp65 Peptivator or 1 µg/ml of Staphylococcal Enterotoxin B (SEB. Sigma-Aldrich, Gillingham, UK) or left untouched. Samples were taken at 1, 4, 6, 16 and 24 hours and stained with APC-conjugated anti-CD3, FITC-conjugated anti-CD4, PerCP-conjugated anti-CD8 and PE-conjugated anti-CD25 (BD Bioscience).

### Separation Using Anti-CD25-Microbeads

CD25-Microbeads (Miltenyi Biotec) were used for both the isolation and depletion of CD25+ cells dependent upon the experiment being performed. For the isolation of antigen-specific T cells following CMVpp65 stimulation, cells were labelled with CD25-microbeads after 16 hour incubation. Labelling was performed for 15 minutes using 10 µl of microbeads per 10^7^ cells in 90 µl of CliniMACS buffer. Following incubation and washing the cell suspension was enriched using MS columns on a MiniMACS (all Miltenyi Biotec). For CD25 depletion experiments cells were labelled using 20 µl of microbeads per 10^7^ cells in 80 µl of CliniMACS buffer. Depletions were performed using LD columns and sorted on a VarioMACS separator (both Miltenyi Biotec). All incubation steps were performed at 4–8°C in the dark. Both enriched and depleted cells were stained with CD3-APC, CD4-FITC, CD8-PerCP, CD69-APC Cy7 (All BD Bioscience) and CD25-PE (Miltenyi Biotec).

### Identification of T Regulatory Cells by FoxP3 Staining

1×10^6^ PBMCs from pre stimulation, CMVpp65 stimulated and CD25 positive fractions were stained with CD3-APC, CD25-PE, CD4-APC Cy7 and CD8-PerCP then fixed and permeabilized using the FoxP3 staining kit (BD Bioscience) before staining with either FoxP3-Alexa Fluor 488 monoclonal antibody or matched IgG isotype control. Samples were acquired on the FACScan flow cytometer with a minimum of 50,000 CD4+ events recorded.

### CFSE Based Proliferation Assay

The carboxyfluorecein diacetate succinimidyl ester (CFSE) based suppression assay was used to assess the suppressive capacity of CMV-T isolated through the activation marker CD25 after 16 hour stimulation with CMVpp65 peptides and compared directly against the CD25 negative fraction. PBMCs were labelled with 1 µM of CFSE (CellTrace CFSE, Life Technologies) and cultured in round bottom 96 well plates (NUNC) with a total of 2×10^5^ cells per well. Depending on the number of effector cells available CD25+ CMV-T and CD2− PBMCs were added to the cultures at a ratio of 1∶1 and 1∶2 of CFSE labelled PBMCs to effectors. Cell cultures were stimulated with 1 µg/ml of SEB (Sigma-Aldrich) or 2 µg/ml of purified anti-CD3 antibody (Clone HIT3a, Biolegend, San Diego, CA). In control experiments CFSE labelled and unlabeled PBMCs were cultured alone. Cultures were incubated for 5 days at 37°C/5% CO_2_ before harvesting and cells stained with CD3-APC and CD69-APC Cy7 (Both BD Biosciences) antibodies. Samples were acquired on the FACScan flow cytometer and a minimum of 10,000 CD3+ CFSE+ events recorded. The frequency of suppression was analysed by gating on the CD3+ CFSE+ population and the percentage of undivided cells (CFSE^high^) determined using the CFSE labelled and unlabelled PBMCs control samples. Percentage of suppression was calculated as follows: [100– (%CFSE^low^ of CD3+ in the presence of effectors/%CFSE^low^ of CD3+ in the absence of effectors) x 100].

### Expansion of Antigen-Specific T Cell Lines

After isolation of CD25+ CMV-T, up to 0.25×10^6^ cells were cultured in the presence of 12.5×10^6^ (50∶1) γ-irradiated (30 Gy) autologous PBMCs to act as feeder cells in 24 well plates with RPMI 1640 medium containing 10% human AB serum, 1% antibiotic and supplemented with 10 ng/ml of IL-7 and IL-15 (CellGenix, Freiburg, Germany). Culture medium was replenished every 2–3 days and cells split when necessary. Cells were expanded up to a maximum of 24 days before harvest.

### Re-stimulation of Expanded Antigen-Specific T Cell Lines

Expanded cells were restimulated for a period of 5–6 hours with either CMVpp65 Peptivator loaded autologous PBMCs or untouched autologous PBMCs as a control, all labelled with 1 µM CFSE (Sigma-Aldrich) at a ratio of 5∶1 at a concentration of 1×10^7^/ml in 48 well plates. For analysis of intracellular cytokines, cells were incubated in the presence of anti-CD28 antibody (BD Bioscience) and 1 µg/ml of Brefeldin A (Sigma-Aldrich) added after 2 hours. Cells were fixed and permeabilized using Intrastain (DakoCytomation, Ely, UK) according to the manufacturer’s instructions and stained with CD25-APC, CD4-PerCP either PE-conjugated anti-IL-2, anti-TNF-α, anti-Granzyme B, anti-IL-10 or anti IFN-γ and CD69-APC Cy7 (all BD Biosciences) monoclonal antibodies. For surface staining cells were stained for 15 minutes with CD4-FITC, CD25-PE, CD8-PerCP, CD3-APC and CD69-APC Cy7 (all BD Biosciences) monoclonal antibodies.

### Cytotoxicity Assay

Autologous PBMCs were stimulated with 3 µg/ml of PHA (Sigma) for 24 hours and then 20 U/ml of IL-2 for a further 72 hours (Miltenyi Biotec) at a concentration of 1×10^6^/ml in RPMI 1640 with 10% AB serum, in 24 well flat bottom plates (NUNC). PHA blasts were then used as target cells in the killing assay and loaded with CMVpp65 Peptivator, or left untouched. Loaded target cells were labelled with Calcein-AM (Life Technologies) at a concentration of 10 µM and incubated for 1 hour at 37°C. After four washes in complete medium cells were adjusted to 7×10^4^/ml and added to effector cells at E: T ratios ranging from 20∶1 to 0.5∶1, in triplicate, in U bottom 96 well plates (NUNC). Triplicate wells were also set up to measure spontaneous release (target cells only), maximal release (target cells plus 2% Triton X-100) and medium alone. After incubation at 37°C/5% CO_2_ for 4 hours, 100 µl of supernatant was harvested and transferred into new plates. Samples were measured using a BMG FLUOstar Galaxy microplate fluorescence spectrophotometer (MTX Lab Systems Inc. Vienna, VA) (excitation filter: 485±9 nm: band-pass filter: 530±9 nm). Data were expressed as arbitrary fluorescent units (AFU) and percent lysis was calculated using the formula [(test release-spontaneous release/maximal release-spontaneous release) x 100].

### Statistical Analysis

Analyses were conducted using GraphPad Prism 4.0 (Graph Pad Software Inc. La Jolla, CA). An unpaired t test was used to determine the statistical significance between G-CSF-mobilized and non-mobilized PBMCs which followed a normal distribution. A paired t test was used for analysing the effect of CD25-depeletion on CD25 and FoxP3 expression. Statistical significance was considered to be achieved when *p* was less than 0.05. Data are presented as mean ± SD.

## Results

### Identification and Isolation of CD25+ CMV-T from G-CSF-mobilized and Non-mobilized PBMCs

**Figure 1:**
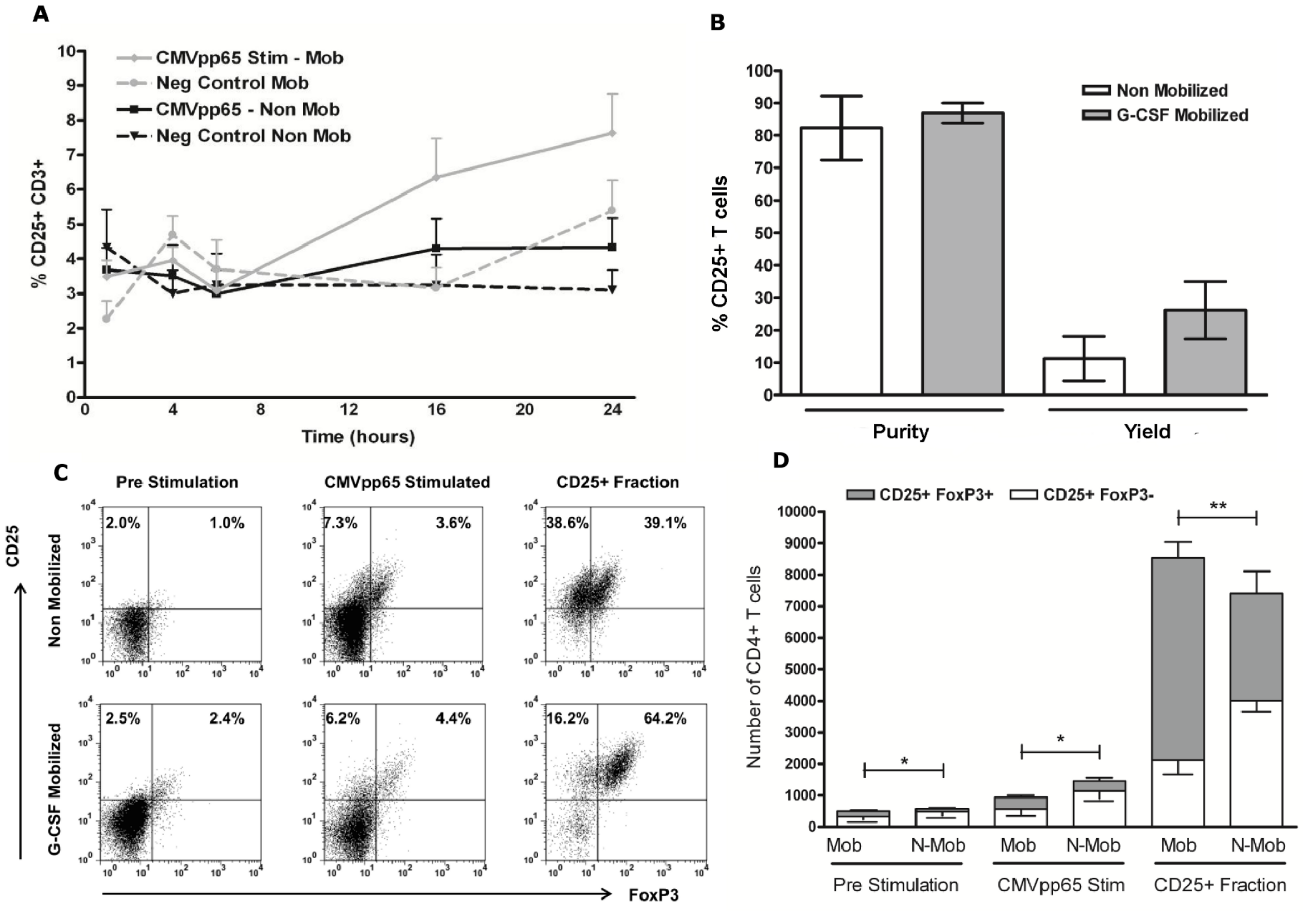
Identification and Isolation of CD25+ CMVpp65-specific T cells in G-CSF-mobilized and non-mobilized PBMCs. G-CSF-mobilized (*n* = 6) and non-mobilized PBMCs (*n* = 5) were stimulated for 24 hours or left untouched (negative control) and samples taken at 1, 4, 6, 16 and 24 hours for analysis of CD25 expression amongst CD3+ T cells (A). Purity and yield of CD25 expressing cells was determined from within the CD3+ population following selection (B). Evaluation of FoxP3 expression in CD4+ CD25+ T cells in G-CSF-mobilized and non-mobilized PBMCs was assessed in pre-stimulated, CMVpp65 stimulated and post CD25+ selection after gating on CD3+ CD4+ T cells (C). Cumulative data of FoxP3 expression was assessed by analysing CD25+FoxP3+ and CD25+FoxP3− populations per 10,000 CD4+ T cells in both G-CSF-mobilized and non-mobilized PBMCs (D). **p<0.01 and *p<0.05 in an unpaired *t* test comparing the absolute number of CD25+FoxP3+ cells. Stim, stimulated; Mob, mobilized; N-Mob, non-mobilized; Neg, negative.

The kinetics of activation induced CD25 expression on CMVpp65 peptide stimulated PBMCs was assessed in G-CSF-mobilized donors to determine the optimal time for maximal expression and compared directly against conventional non-mobilized PBMCs. Baseline CD25 expression was assessed prior to CMVpp65 stimulation amongst CD3+ T cells with no significant difference observed between G-CSF-mobilized (4.5% ±0.59) and non-mobilized (4.3% ±0.59; *p* = 0.83) PBMCs. PBMCs were stimulated over 24 hours, sampled at 1, 4, 6, 16 and 24 hours and analysed for CD25 expression by flow cytometry (Fig. 1A). The mean antigen-dependent expression of CD25 was maximal at 24 hours in G-CSF-mobilized PBMCs (7.63% ±1.12) and was significantly elevated when compared to non-mobilized PBMCs (4.33% ±0.84; *p* = 0.04). Further analysis of CD25 expression in G-CSF-mobilized PBMCs revealed the optimal time of expression to be at 16 hours after subtraction of the unstimulated control when compared with 24 hour stimulation (3.20% ±1.43 vs. 2.25% ±0.76). On the basis of the results from expression kinetics, isolation of CD25+ CMV-T was investigated following 16 hour CMVpp65 peptide stimulation. A single enrichment step was performed using magnetic-bead based cell separation in six G-CSF-mobilized and five non-mobilized healthy CMV-seropositive donors. No significant difference was observed in CD25+ purity (86.9% ±3.1 vs. 82.3% ±9.9; *p* = 0.32) or yield (26.1% ±8.8 vs. 11.3% ±6.9; *p* = 0.12) when comparing G-CSF-mobilized and non-mobilized PBMCs (Fig. 1B). Yield was defined as the absolute number of CD25+ T cells in the positive fraction as a proportion of the absolute number of CD25+ T cells in the pre-sort sample. Analysis of CD3+ T cell subsets in CD25 positive fractions revealed a predominantly CD4+ population in both G-CSF-mobilized (97.6% ±1.8) and non-mobilized (92.3% ±4.9) PBMCs.

### FoxP3 Expression in CD25+ CMV-T

To evaluate the suitability of CD25 as a target for isolation of CMV-T from G-CSF-mobilized PBMCs, the level of Treg enrichment was determined by analysing the proportion of CD25+FoxP3+ cells amongst CD4+ cells at three time points: (1) pre-CMVpp65 stimulation; (2) post-CMVpp65 stimulation; and (3) post-CD25 enrichment. Figure 1C illustrates a representative experiment in both a G-CSF-mobilized and non-mobilized donor showing the increased level of CD25+FoxP3+ expression present in G-CSF-mobilized PBMCs post CD25 enrichment. Figure 1D summarizes experiments from six G-CSF-mobilized and five non-mobilized donors where analysis revealed that the proportion of FoxP3+ cells contained within the CD4+CD25+ population was significantly increased in G-CSF-mobilized PBMCs in resting PBMCs (41.35% ±8.41 vs. 17.5% ±6.5; *p* = 0.04) and following CMVpp65 stimulation (42.79% ±4.07 vs. 20.84% ±5.46; *p* = 0.012), when compared with non-mobilized PBMCs. The proportion of CD25+FoxP3+ cells after CD25 enrichment was also shown to be significantly increased in G-CSF-mobilized (73.64% ±4.88) vs. non-mobilized (43.82% ±5.64; *p* = 0.004) PBMCs.

### Assessment of Suppressive Capacity of CD25+ enriched CMV-T in G-CSF-mobilized PBMCs

**Figure 2:**
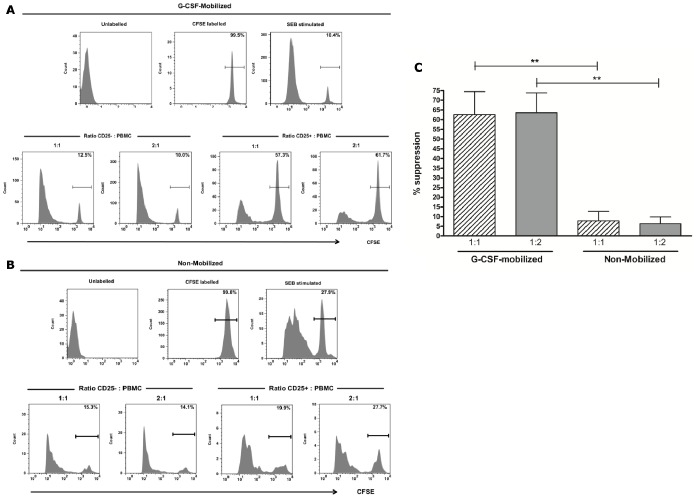
Suppression of autologous PBMCs by CMV-stimulated CD25+ cells isolated following CMVpp65 stimulation. Suppressive capacity was assessed in CD25-positive and CD25-negative fractions after magnetic selection following CMVpp65 stimulation between G-CSF-mobilized (A) and non-mobilized (B) donors. CFSE-labelled PBMCs were cultured at two ratios in the presence of SEB for 5 days. Cumulative data in G-CSF-mobilized (*n* = 5) and non-mobilized (*n* = 5) donors are shown at a ratio of 1∶1 (diagonal bars) and 1∶2 (shaded bars) PBMCs to CD25+ selected T cells after calculating percentage of suppression as indicated in the Material and Methods section. Data are presented as mean values with standard deviation (C). Unlabelled, PBMCs alone (CFSE) and PBMCs with SEB were cultured as experimental controls. **p<0.01, in an unpaired *t* test.

Having demonstrated that CD25+ cells enriched after CMVpp65 stimulation from G-CSF-mobilized PBMCs contain a significant proportion of FoxP3-expressing cells, their suppressive activity was assessed using a CFSE-based T cell proliferation assay. The suppressive capacity of CD25+ T cells enriched after CMVpp65 stimulation was compared to the CD25− fraction, in both G-CSF-mobilized (*n* = 5) and non-mobilized PBMCs (*n* = 5). Both fractions were cultured at ratios of 1∶1 and 2∶1 and in some experiments at 5∶1 (effectors: responders), with CFSE-labelled PBMCs, in the presence of the superantigen SEB. Figure 2A illustrates a representative experiment from a G-CSF-mobilized donor, where T cell proliferation in the presence of CD25− cells was similar to PBMCs alone (10.4% undivided CD3+ T cells) at both 1∶1 (12.5% undivided CD3+ T cells) and 2∶1 (10.0% undivided CD3+ T cells). In contrast, T cell proliferation was greatly reduced when CFSE-labelled PBMCs were cultured in the presence of CD25+ CMV-T at both 1∶1 (57.3% undivided CD3+ T cells) and 2∶1 (61.7% undivided CD3+ T cells), highlighting a strong suppressive capacity. Suppression of T cell proliferation by CD25+ CMV-T isolated from non-mobilized PBMCs was shown to be reduced when compared with G-CSF-mobilized PBMCs. Figure 2B shows the results from a representative experiment using non-mobilized PBMCs, where proliferation of CFSE-labelled PBMCs in response to SEB stimulation was comparable when cultured with CD25− and CD25+ cells at both ratios of 1∶1 (15.3% vs. 19.9% undivided CD3+ T cells) and 2∶1 (14.1% vs. 27.7% respectively). Cumulative analysis of CD25+ enriched T cells following CMVpp65 stimulation revealed a significant difference in the mean suppressive capacity of G-CSF-mobilized (*n* = 5) compared to non-mobilized (*n* = 5) PBMCs (Fig 2C). G-CSF-mobilized PBMCs were highly suppressive of autologous PBMCs proliferation following SEB stimulation at both ratios of 1∶1 (62.7% ±11.75 suppression) and 2∶1 (63.58% ±10.27). This was significantly reduced in non-mobilized PBMCs at both ratios of 1∶1 (7.90% ±4.79; *p* = 0.007) and 2∶1 (6.37% ±3.48; *p* = 0.002) PBMCs.

### Depletion of CD25-expressing Cells in Resting G-CSF-mobilized PBMCs

**Figure 3:**
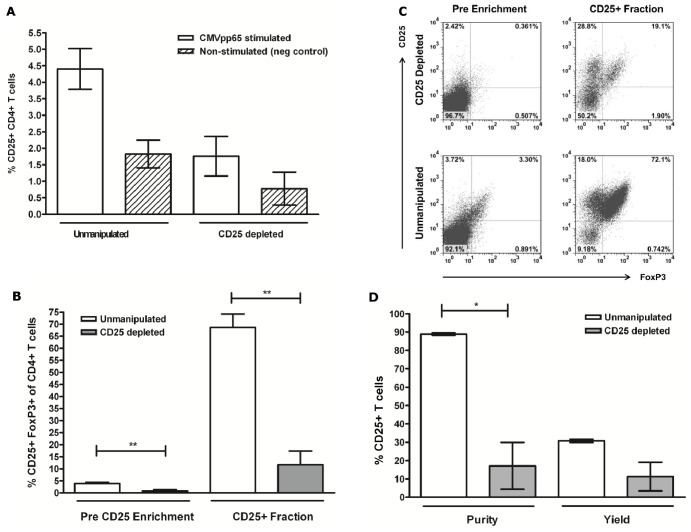
Effect of CD25 immunomagnetic depletion on CD25+ CMVpp65 CMV-T and CD25+ FoxP3+ Tregs. CD25 expression was assessed in paired unmanipulated and CD25-depleted G-CSF-mobilized PBMCs following CMVpp65 peptide stimulation (A). FoxP3 expression was analysed both pre and post CD25-enrichment amongst CD4+ T cells (B–D) and CD25 purity and yield determined following CD25 enrichment (D). **p<0.01 and *p<0.05 in a paired *t* test. Neg, negative.

To determine the role of Tregs in CD25+ T cell responses to CMVpp65 peptide stimulation following isolation from G-CSF-mobilized PBMCs, we depleted CD25 expressing cells prior to stimulation. CD25-depleted G-CSF-mobilized PBMCs were compared to unmanipulated PBMCs to assess the impact on CD25 purity, yield and FoxP3 expression following CMVpp65 stimulation and CD25 magnetic enrichment. Mean CD25 expression amongst CD3+ T cells in CD25 depleted G-CSF-mobilized PBMCs, pre stimulation, was 0.57% ±0.13 compared to 2.85% ±0.69 (*p* = 0.05) in the unmanipulated paired samples. CD25 expression post CMVpp65 stimulation amongst CD4+ T cells (Fig. 3A) was reduced when comparing unmanipulated (4.40% ±0.61) and CD25-depleted G-CSF-mobilized PBMCs (1.75% ±0.59). CD25+ FoxP3 expression was assessed pre and post CD25 enrichment to determine the impact of CD25−depletion on the significant proportion of Tregs identified in CD25+ fractions following immunomagnetic CD25-enrichment. CD25-depletion significantly reduced FoxP3 expression (Figs. 3B–C) both pre CD25 enrichment (3.88% ±0.51 vs. 0.74% ±0.55; *p* = 0.002) and post CD25 enrichment (68.60% ±5.56 vs. 11.59% ±5.75; *p* = 0.001) in G-CSF-mobilized PBMCs. Assessment of CD25 expression following CMVpp65 stimulation and CD25 enrichment revealed that CD25-depeletion had a significantly negative impact on CD25 purity when comparing depleted versus unmanipulated (17.08% ±12.78 vs. 88.91% ±0.57 respectively; *p* = 0.03) in G-CSF-mobilized PBMCs (Figure 3D). However, analysis of mean CD25+FoxP3− T cells amongst CD4+ T cells following enrichment revealed no significance (*p*>0.05) between CD25-depleted (16.93% ±6.34) and unmanipulated (22.20% ±7.18) G-CSF-mobilized PBMCs, suggesting that CD25 depletion does not remove CMVpp65-specific T cells.

### Expansion of in-vitro Expanded CD25+ CMV-T and Assessment of CMV Specificity

**Figure 4:**
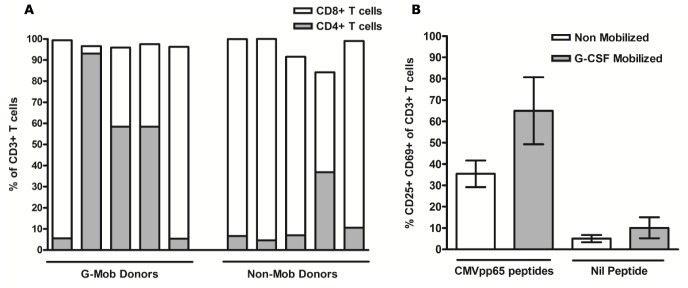
Expansion of CD25+ CMVpp65-specific T cells in short term culture. 0.25×10^6^ CD25+ T cells were expanded in culture for up to 24 days in the presence of IL-7, IL-15 and 12.5×10^6^ irradiated autologous feeder cells (ratio of 1∶50) in G-CSF-mobilized and non-mobilized donors. CD25+ expanded cells were analysed for the proportion of CD8+ (open bars) and CD4+ (grey bars) T cells amongst CD3+ T cells in both G-CSF-mobilized and non-mobilized donors (A). Expanded cells were assessed for co-expression of CD25 and CD69 following CMVpp65 re-challenge in both G-CSF-mobilized (*n = 5*) and non-mobilized (*n = 5*) expansions (B). Expanded cells were also challenged with control feeders (without CMVpp65 peptides). G-Mob, G-CSF-Mobilized; Non-mob, Non-Mobilized.

CD25+ CMV-T were cultured for up to 24 days in complete medium supplemented with IL-7 and IL-15 in the presence of autologous irradiated feeder cells. The mean proliferative capacity of G-CSF-mobilized CD25+ (90.3-fold expansion ±30.0; *n* = 5) was reduced (*p* = 0.07) when compared with non-mobilized CD25+ CMV-T (237.0-fold expansion ±63.8; *n* = 5) Expanded cells from G-CSF-mobilized PBMCs revealed a higher proportion of CD4+ T cells compared with non-mobilized PBMCs (44.13% ±17.0 vs. 13.9% ±5.9) and a reduction in the proportion of CD8+ T cells (53.0% ±17.3 vs. 79.1% ±8.7). In two of the five CD25 expansion experiments performed from G-CSF-mobilized donors, the CD8+ subset at day 22 accounted for >90% of CD3+ T cells compared with <5.5% CD4+, which was contradictory to the results seen in the remaining three CD25 expansions and illustrates a high level of donor variability in cultured cells, not seen in the non-mobilized setting (Fig. 4A). Cultures showed specificity for CMVpp65, determined by up-regulation of CD25+ and CD69+ expression following re-challenge with autologous CMVpp65-loaded PBMCs in contrast to re-challenge with autologous PBMCs without peptide (Fig. 4B). The mean level of CD25+ CD69+ co-expression in CD25+ expanded CMV-T from G-CSF-mobilized (64.9% ±15.7) PBMCs was elevated when compared to non-mobilized (35.4% ±6.2) PBMCs. In control experiments, re-challenge with autologous PBMCs alone produced a low mean level of CD25+ CD69+ co-expression in CD25+ expanded CMV-T from G-CSF-mobilized (10.0% ±4.9) and non-mobilized (5.0% ±1.7) PBMCs.

### CD25+ Expanded Cells Synthesize and Secrete an Effector Cytokine Repertoire and Effectively Lyse CMVpp65 Targets

**Figure 5:**
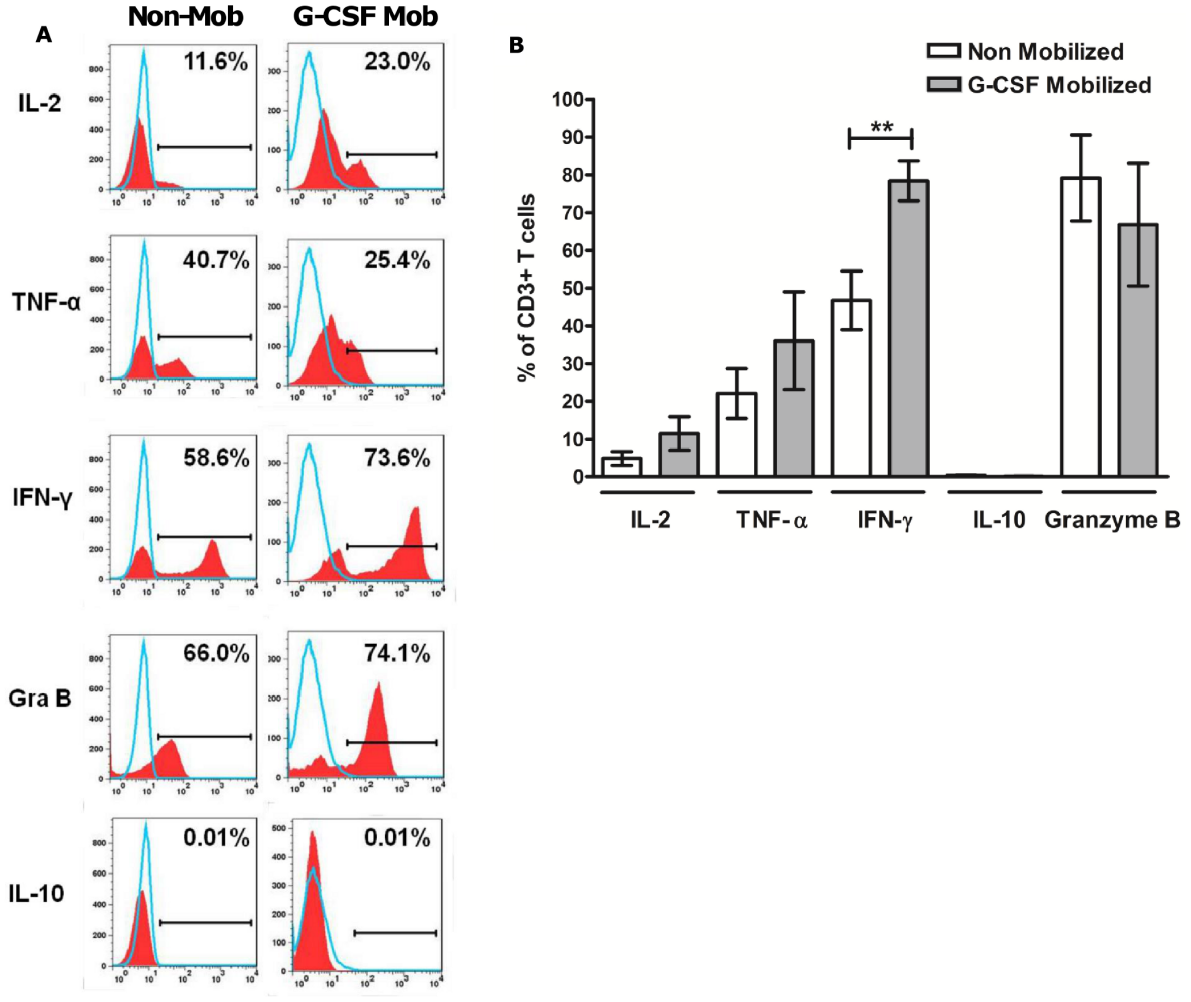
Qualitative and quantitative assessment of cytokine secretion by expanded cells after antigenic re-challenge. Expanded CD25+ cells isolated from both G-CSF-mobilized and non-mobilized donors were stimulated with CMVpp65 peptide loaded autologous PBMCs in the presence of Brefeldin A and purified anti CD28-antibody. Histograms illustrate CMVpp65 rechallenge experiments in representative CD25+ expansions from a G-CSF-mobilized and non-mobilized donor, analysed for IL-2, TNF-α, IFN-γ, Granzyme B and IL-10. Open peaks represent isotype matched controls (A). The combined assessment in G-CSF-mobilized (*n* = 5) and non-mobilized (*n* = 5) donors are summarized (B). **p<0.01, in an unpaired *t* test. Gra, granzyme; Mob, mobilized.

Antigen specificity was assessed through intracellular cytokine staining (ICS) following CMVpp65 re-challenge (G-CSF-mobilized, *n* = 5; non-mobilized, *n* = 5). CD25+ expanded cells revealed low to undetectable mean levels of IL-10 secretion after CMVpp65 re-challenge from both G-CSF-mobilized (0.11% ±0.05) and non-mobilized (0.41% ±0.10) PBMCs. Expanded cells synthesized and secreted low levels of IL-2 and significant levels of TNF-α, IFN-γ and Granzyme B (Figs. 5A–B), indicating that cells possessed the effector molecules necessary for cytotoxic activity. Cytokine secretion was comparable between G-CSF-mobilized and non-mobilized CD25+ expanded cells, apart from IFN-γ where G-CSF-mobilized CD25+ CMV-T revealed a significant increase (*p* = 0.009) in secretion compared with non-mobilized CD25+ CMV-T (78.4% ±5.2 vs. 46.7% ±7.7). In experiments where CD25+ expanded cells were re-challenged with unstimulated or CMV IE-1 peptide loaded autologous PBMCs minimal cytokine secretion was observed (data not shown).

**Figure 6:**
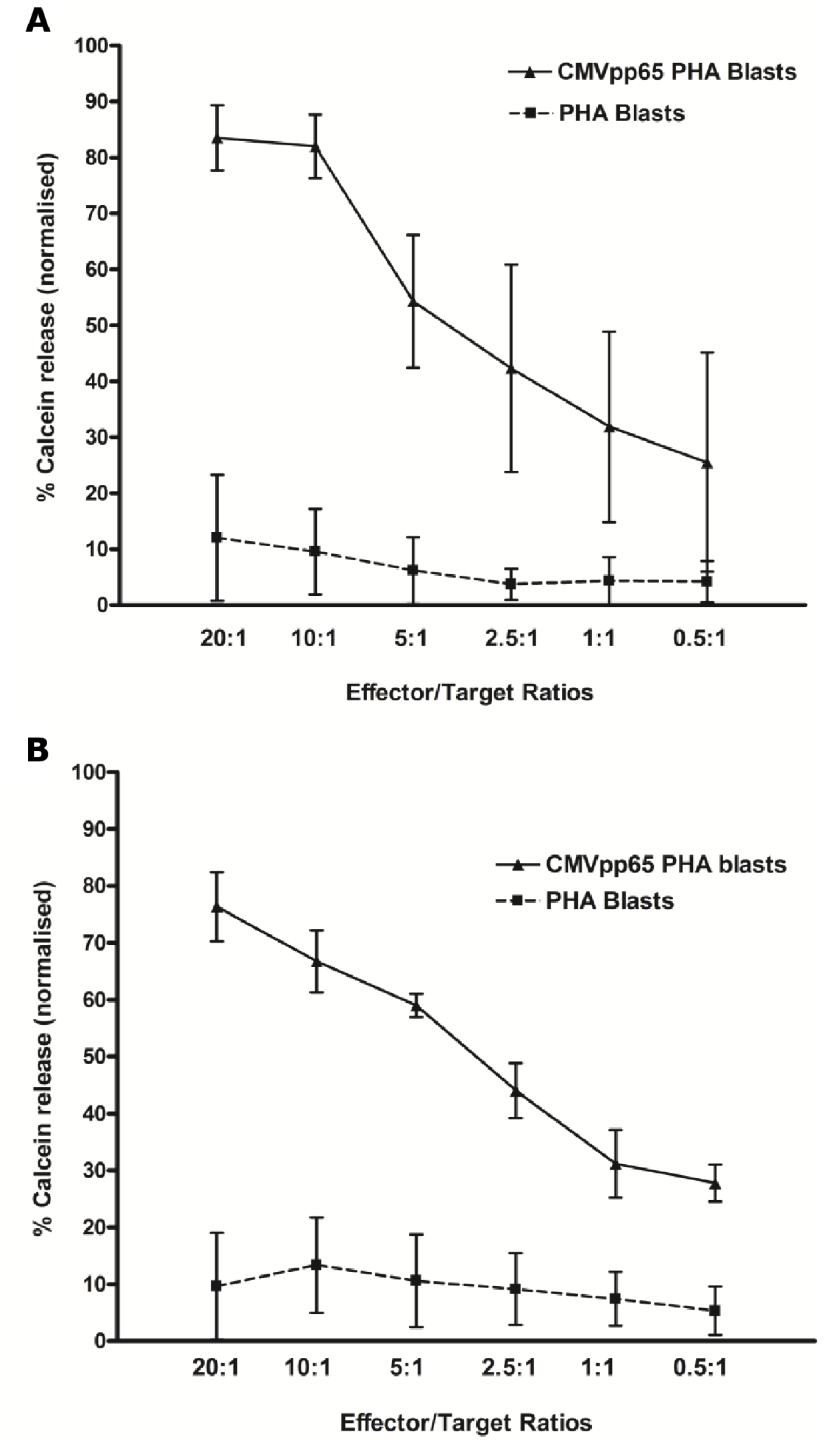
Cytotoxicity of CD25+ expanded CMV-T. Specific lysis of autologous PHA blasts loaded with CMVpp65 peptides at E:T ratios from 20∶1 to 0.5∶1 was determined using fluorescent dye Calcein-AM cytotoxicity assay in CD25+ expanded cells. Graphs show pooled data from all cytotoxicity experiments in G-CSF-mobilized (A; *n* = 4) and non-mobilized (B; *n* = 4) CD25+ expanded CMV-T.

Cytotoxic activity was evaluated using autologous PHA blasts loaded with CMVpp65 peptides and labelled with Calcein-AM dye as targets. CD25+ expanded cells from both G-CSF-mobilized (*n* = 4) and non-mobilized (*n* = 4) PBMCs effectively lysed CMVpp65 targets at all E: T ratios in a dose-dependent manner (Figs. 6A–B), with minimal lysis of PHA blasts without CMVpp65 peptides. Comparison between G-CSF-mobilized and non-mobilized CD25+ CMV-T revealed no significant difference in lysis of CMVpp65 PHA blasts at any of the E:T ratios.

### Assessment of FoxP3 Expression and Suppressive Capacity of CD25+ Expanded CMV-T from G-CSF-mobilized PBMCs

**Figure 7:**
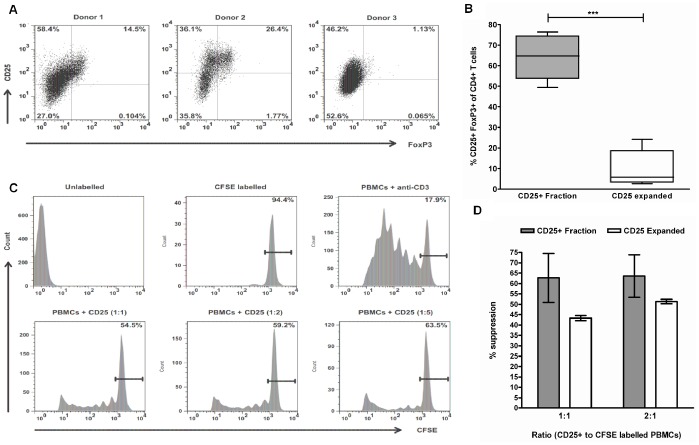
Assessment of FoxP3 expression and suppressive capacity of CD25+ expanded cells in G-CSF-mobilized PBMCs. FoxP3 expression was assessed in G-CSF-mobilized CD25+ expanded cells after 14–24 days in culture. FACS plots illustrate results in 3 expansion experiments (A). FoxP3 expression was compared between CD25+ expanded cells (*n* = 5) and CD25+ fractions following magnetic enrichment (*n* = 5) (B). Suppression of CFSE-labelled PBMCs proliferation by CD25+ expanded cells (*n* = 3) was determined at three ratios in the presence of anti-CD3 antibody cultured for 5 days (C). PBMCs alone (CFSE) and PBMCs incubated with anti-CD3 antibody were cultured as experimental controls. Assessment of suppressive capacity was determined by comparing CD25+ expanded cells with CD25+ fractions (D). ***p<0.0001, in an unpaired *t* test.

Previous experiments identified that CD25+ cells enriched from G-CSF-mobilized PBMCs following CMVpp65 peptide stimulation contained a significant proportion of FoxP3 expressing cells. Assessment of FoxP3 expression in CD25 expanded cells after short term culture is illustrated in three expansions (Fig. 7A) and revealed a significant reduction (*p* = <0.0001) in expression compared to pre-expansion. Mean FoxP3 expression amongst CD4+ T cells after culture was 9.89% ±4.02, compared to 64.24% ±4.88 at pre-expansion (Fig. 7B). The suppressive capacity of CD25+ expanded cells from G-CSF-mobilized PBMCs were assessed in a 5-day CFSE proliferation assay (Fig. 7C) as previously described. G-CSF-mobilized CD25+ expanded cells were shown to retain their suppressive capacity at all ratios at a level comparable with CD25 enriched cells post CMVpp65 stimulation (Fig. 7D). Mean suppression of autologous PBMCs when co-cultured at 1∶2 with CD25+ expanded cells was 51.35% ±1.05 compared with 63.58% ±10.27 with CD25+ enriched cells.

## Discussion

CD25 expression has been reported as an optimal marker for the identification of antigen-specific T cells following peptide stimulation [17]–[21], enabling the magnetic enrichment of activated anti-viral T cells that would otherwise be undetectable using current IFN-γ secretion methods. Generation of anti-viral T cells has been largely restricted to non-mobilized steady state leukapheresis or a single blood draw for manufacture. More recently, studies have investigated the use of G-CSF-mobilized PBMCs from the original aHSCT graft as a potential source for CMV-T manufacture [31]; [32]. Here, we expand on these studies to investigate the activation marker CD25 as a possible target and investigate the nature of CD25+ enriched cells following CMV peptide stimulation with regard to Treg characterisation, due to their increased number following G-CSF-mobilization [36].

We have shown that CD25 expression is up-regulated in G-CSF-mobilized PBMCs after CMVpp65 peptide stimulation at a level comparable to that seen in non-mobilized PBMCs and that magnetic enrichment of CD25 expressing cells results in a high level of purity. However further analysis revealed that the CD25 positive fractions isolated from G-CSF-mobilized PBMCs co-expressed FoxP3, a population that was largely absent in the CD25 positive fraction isolated from non-mobilized PBMCs. Functional studies revealed they were highly suppressive *in vitro*, demonstrating a significant population of Tregs. Several studies have indicated the existence of anti-viral Tregs, revealed following antigenic stimulation [37]–[42] and indeed Ltjens and colleagues concluded that CD4+CD25+CD127−FoxP3+ Tregs contained a population of CMV-specific T cells [38], which is consistent with the results seen here. This suggests that CMVpp65 stimulation in G-CSF-mobilized PBMCs induces a population of antigen-specific CD25+ Tregs that are able to recognize CMVpp65 peptides and possibly proliferate in response to engagement of their cognate antigen. Support for this hypothesis is evident in a murine model of *Leishmania* infection, in which Tregs proliferated in response to recognition of parasite derived antigen [40]. Previous studies [21] have suggested that the co-infusion of Tregs and anti-viral T cells could be advantageous in terms of an immunotherapy that inhibits GvHD on one hand and promotes ant-viral immune reconstitution on the other. It has also been argued that it is unlikely that infused Tregs will prevent proliferation of virus-specific T cells; a hypothesis supported by studies in mice demonstrating co-infusion of Tregs and conventional T cells (Tcons) enhances virus-specific immune reconstitution and protected mice from lethal CMV infection in the HSCT setting [43]. Clinical studies have since demonstrated that early infusion of freshly isolated donor Tregs followed by Tcons at the time of full-haplotype-mismatched aHSCT prevented GvHD in the absence of any post-transplant immunosuppression and improved immunity to opportunistic pathogens [44]. However in this study Tregs were generated from steady-state leukapheresis and did not use viral antigen-specific Tregs which appear to be preferentially generated from G-CSF-mobilized PBMCs.

In the context of manufacturing CMV-T for adoptive immunotherapy the emergence of a population of CD25+FoxP3+ cells from G-CSF-mobilized PBMCs with a highly suppressive capacity suggests that CD25 will be an unsuitable target for CMV-T isolation. We therefore investigated the impact of depleting CD25-expressing cells prior to CMVpp65 peptide stimulation in order to eliminate the CD25+FoxP3+ cells identified following enrichment from G-CSF-mobilized PBMCs. Depletion of CD25 expressing cells in G-CSF-mobilized PBMCs, prior to CMVpp65 peptide stimulation, resulted in a reduction in CD25 expression post stimulation which in turn had a negative impact on both yield and purity following CD25 enrichment, confirming previous studies by Melenhorst and colleagues in non-mobilized PBMCs (39). The reduction in CD25 expression is partly explained by the elimination of contaminating Tregs prior to CMVpp65 stimulation, as indeed was the aim, but could also be attributed to a population of CMV-T that are in an activated state and express CD25 prior to CMVpp65 stimulation *in vitro*. Although a significant reduction in FoxP3 expression was observed in CD25-depleted PBMCs post-CMVpp65 stimulation, FoxP3 expression post-CD25 enrichment was still detectable. These results suggest either the emergence of newly expressing FoxP3 cells or persistence of FoxP3+CD25– cells following the depletion step and comparable to results seen in non-mobilized cells [39]–[42] and supports the concept of CMV-specific Tregs.

To determine the functionality of CD25+ CMV-T and the possible impact of Tregs on the anti-CMV response, we analysed whether these cells were capable of secreting inflammatory cytokines in response to antigenic re-challenge following short-term culture. CD25+ expanded cells from G-CSF-mobilized PBMCs secreted high levels of IFN-γ and Granzyme B, and to a lesser extent TNF-α and IL-2, in the absence of IL-10. This cytokine profile is in keeping with anti-viral CTL rather than antigen-specific Tregs, which was confirmed by their ability to lyse autologous CMV-pulsed target cells. Further analysis of FoxP3 expression also revealed a significant reduction following expansion. Nonetheless expanded CD25+ CMV-T retained a capacity to suppress polyclonal T cells proliferation suggesting the presence of a population of FoxP3– IL10– Tregs. However it must be noted that a limitation of this study was the failure to procure paired donor samples before and after G-CSF-mobilization for subsequent comparison of both the CMV immune response and Treg numbers and potency. The use of paired samples could have allowed for stronger statistical analysis to be made when analysing the effect of G-CSF-mobilization given the knowledge of high donor variability that exists, in terms of the CMV immune response.

Although FoxP3 generally identifies natural thymus-derived Tregs, adaptive Tregs may or may not express this transcription factor [45]. Studies have also indicated that FoxP3 expression can be induced in human CD4+ effector T cells after activation as a normal consequence of CD4+ T cell activation [42]–[46] and that IFN-γ and IL-2 production are not suppressed in FoxP3+ effector T cells [47], [48], thus bringing into doubt its validity as an exclusive marker of Tregs. The characterisation of the pool of CD25+FoxP3+ Tregs identified post CD25 enrichment following CMVpp65 stimulation and the subsequent loss of FoxP3 expression following short-term culture highlights the need for future studies. Most notably to extensively characterise these highly suppressive cells with regard to disseminating between inducible/adaptive and antigen-specific subsets of Tregs, that may be committed to regulating specific arms of the immune response.

In conclusion our data indicates that CD25 enrichment post CMV stimulation in G-CSF-mobilized PBMCs results in the simultaneous generation of a functional mixed population of CMV-T and Tregs and outlines a potential therapeutic strategy for the treatment of both GvHD and CMV reactivation following HSCT. The use of G-CSF-mobilized PBMCs represents a feasible approach to alleviating the many problems incurred with successive donations and procurement of cells from unrelated donors and thereby simplifying the clinical application of cellular therapy manufacture in the aHSCT setting. Questions remain as to whether these cells are capable of both conferring protection from CMV and minimising the GvH response *in vivo* and whether the infused Treg population will prevent the persistence and proliferation of CMV-T, both of which can only be answered by clinical trials utilizing this approach, in recipients of aHSCT.
